# Theory-Based Social and Non-Social Engagement Features in Smoking Cessation Mobile Apps: A Content Analysis

**DOI:** 10.3390/ijerph18179106

**Published:** 2021-08-29

**Authors:** Qinghua Yang

**Affiliations:** Bob Schieffer College of Communication, Texas Christian University, Fort Worth, TX 76129, USA; q.yang@tcu.edu

**Keywords:** mobile applications, engagement features, smoking cessation, content analysis

## Abstract

Despite the ubiquity of smartphone ownership and the increasing integration of social engagement features in smoking cessation apps to engage users, the social and non-social engagement features that are present in current smoking cessation apps and the effectiveness of these features in engaging users remain understudied. To fill the gap in the literature, a content analysis of free and paid smoking cessation mobile apps was conducted to examine (a) the presence of social features (i.e., social support, social announcement, and social referencing) and non-social engagement features (e.g., personal environmental changes, goal setting, progress tracking, reinforcement tracking, self-monitoring, and personalized recommendations) and (b) their relationships with user engagement scores measured by the Mobile App Rating Scale. In this study, 28.2% of the smoking cessation apps enable social announcement and 8.1% offered the social support feature. Only two apps provided a social referencing feature (1.3%). No app included reinforcement tracking, with the percentage of other non-social engagement features ranging from 9.4% to 49.0%. Social support (*β* = 0.30, *p* < 0.001), social announcement (*β* = 0.21, *p* < 0.05), and social referencing (*β* = 0.18, *p* < 0.05) were significant predictors of user engagement. Regarding the non-social engagement features, personal environment changes (*β* = 0.38, *p* < 0.001), progress tracking (*β* = 0.18, *p* < 0.05), and personalized recommendations (*β* = 0.37, *p* < 0.001) significantly predicted user engagement. The findings not only contribute to the mobile communication literature by applying and extending the theory-based mobile health apps engagement typology, but also inform the future architecture design of smoking cessation mobile apps.

## 1. Introduction

The number of smartphone owners in the U.S. has been rapidly growing, increasing from 35% in 2011 to 77% in 2018 [1], and the use and capabilities of these mobile devices will likely continue to expand. The widespread adoption of smartphones has allowed for ubiquitous access to and use of health-related mobile applications (apps) on the part of consumers. For instance, Krebs and Duncan [2] documented from a national survey that 58% of U.S. mobile phone owners have downloaded at least one health-related mobile app, among whom 42% had downloaded more than five health-related apps. The popularity of health-related mobile apps among users also inspired the delivery of mobile application-based interventions, also referred to as mobile health (or mHealth), by health care providers and researchers. A recent meta-analytic review analyzed 64 mHealth interventions and reported a small but significant overall effect size, indicating the general effectiveness and promise of mobile phone interventions across health topics [3].

Mobile health apps have been focusing on tobacco use as one primary health issue [4]. Although cigarette smoking has remarkably declined over the past six decades, it remains the leading cause of preventable death in the U.S., claiming over 480,000 lives annually, including more than 41,000 deaths due to secondhand smoking [5]. Compared to mass media or in-person smoking cessation programs, mobile apps offer several unique advantages, such as maintaining anonymity and reduced stigma, interactive and tailored features that help engage smokers, daily reminders and progress tracking that improve self-monitoring, few geographical constraints, and higher cost-effectiveness in reaching a large and diverse population [6,7]. While past studies have highlighted the potential of using mobile apps for successful smoking cessation, their features and efficacy remain unclear [8], calling for more empirical research to better understand the effectiveness of smoking cessation mobile apps [9,10].

User engagement, a “combination of attention, interest, and enjoyment” [11] (p. 386), was identified as a key mediating variable in regards to the effectiveness of new-media-based health interventions [12,13], and has been shown to promote health behaviors [14,15]. New media that include interactive components, such as social features, promote engagement and can make users feel more connected [16]. However, only a small proportion of apps have been developed in conjunction with social features [17], and few studies have examined their functions and effectiveness in engaging users. One content analysis identified 31 smoking cessation apps that had social media capabilities [17], but did not specify their functions or engagement strategies, leaving the presentations and effects of social engagement features in mobile health apps unclear.

Besides social engagement features (e.g., social announcement, social referencing, and social support), there also exist multiple forms of non-social engagement features (e.g., goal setting, progress tracking, self-monitoring, and personalized recommendations) for mobile apps [18], which are based on social-psychological theories. However, given the paucity of scholarship focusing on smoking-related mHealth, which specific features lead to higher user engagement for smoking cessation apps remains unknown. To fill the gap in the literature and shed light on mHealth theories, a content analysis of smoking cessation apps was conducted, aiming to understand the social and non-social engagement features, and how they influence user engagement. 

### 1.1. Smoking Cessation Mobile Apps

As the leading cause of preventable morbidity and mortality in the U.S., tobacco smoking can cause cancer almost anywhere in the human body and is a risk factor for cardiovascular diseases, stroke, and infertility [5]. Although the large-scale anti-smoking campaigns in recent years, such as The Real Cost by the Food and Drug Administration, were found effective in preventing tobacco use [19], those ready-made messages distributed in the one-to-many manner make customization and interactivity impossible. In addition, stigma has been associated with smoking, especially among young, female, and pregnant smokers, which makes face-to-face counseling and behavioral interventions difficult [6].

Mobile technologies, which include monitoring, tracking, real-time and personalized feedback, and interactive components, have transformed health care, by enhancing active engagement, improving health outcomes, and substantially lowering costs [20]. Text-messaging-based mobile health programs, for instance, offering both asynchronous and synchronous communication with support networks and reducing geographic, cost, and scheduling barriers, have showed effectiveness in improving user engagement [21,22]. For instance, Text2Quit, an interactive and personalized mHealth smoking cessation program with text messaging serving as the central component, was found engaging among college students [23]. However, compared to the limited functionality and modality of text messaging-based programs, mobile apps provide several additional benefits for smoking cessation, such as a higher level of medium-centered (e.g., video games) [24] and human-centered interactivity (e.g., forums and online support groups) [25], as well as customized advices for smokers, self-monitoring, progress tracking, and regular reminders [4,7], and therefore, they have attracted an increasing level of scholarly attention.

Previous content analyses of mobile apps for smoking cessation have explored adherence to national guidelines, the presence of self-determination theory, the price and market of the apps, the content and functions (e.g., information and function), and user-rating and downloads [17,26,27,28]. Specifically, Abroms and colleagues indicated that few apps (a) adhered to the U.S. Public Health Service’s Clinical Practice Guidelines for Treating Tobacco Use and Dependence (CPG-TTUD; see [29] for review) and (b) recommended users to proven treatments (e.g., pharmacotherapy and quit line) [26,27]. In the same vein, earlier smoking cessation mobile apps have primarily focused on simplistic tools, such as a calculator or calendar, while sparingly leveraged tailoring features (i.e., interactive, proactive, and responsive features), albeit positively associated with mHealth app popularity and user-rated quality [28]. Under the framework of self-determination theory, Choi et al. pointed out that existing smoking cessation apps have been limited in features that sufficiently stimulate autonomous motivation for long-term cessation, with extrinsic goals (e.g., money and appearance) being more emphasized, through primarily gain-framed messages, than intrinsic goals (e.g., health) [17]. Despite their pioneering efforts, no study has yet systematically examined social and non-social engagement features in smoking cessation mobile apps, leaving their prevalence and effectiveness in engaging smokers unknown.

### 1.2. Engagement Features of Health-Related Mobile Apps

Health-related mobile apps provide a wide range of opportunities for engagement among users. User engagement, as “the theoretical framework used by the app to promote the desired health outcome” [18] (p. 2), is fundamental for the success of smoking cessation apps [30], since actively engaging users benefit most from the programs [15]. However, in real practices, users’ engagement or interactivity usually decreases over time, which poses a threat to the effectiveness of smoking cessation apps [31].

To enhance users’ longitudinal engagement, several theoretical frameworks have been applied, such as social support theory [32], social comparison theory [33], social cognitive theory [34,35], and self-determination theory [17,36], to strategize the mobile design. One typology that was proposed specifically for health-related mobile apps by Sama and colleagues has been primarily used to understand mobile app user engagement [18]. According to Sama et al., types of engagement can be classified into two major categories: social and non-social engagement features [18].

#### 1.2.1. Social Engagement Features

Three features enabled by mHealth apps are related to social networking [18]. First, social support, rooted in the social support theory [32], provides a means by which users can connect to others via groups online or in person for encouragement, problem solving, solidarity, etc. Social presentation or announcement, involving a downward social comparison and a sense of achievement [33], allows smokers to share their accomplishments (e.g., number of days on cessation) with other users in-app or via social media. Social referencing, involving primarily an upward social comparison, provides a reference point to users so that they can see how great their accomplishments are compared to similar other users.

#### 1.2.2. Non-Social Engagement Features

Besides the three social engagement features, another six major non-social features are supposedly prevalent among mHealth apps: (a) changing personal environment, (b) goal setting, (c) progress tracking, (d) reinforcement tracking, (e) self-monitoring, and (f) personalized recommendations [18,37]. First, changing personal environment allows users to alter their environment, such as background picture or calming music for stress relief. While the goal setting feature allows users to set a goal (e.g., a desired weight), the progress tracking feature provides secondary goals to assist users in achieving their ultimate goals and the reinforcement tracking gives some form of feedback to users based on the health data recorded in the app through a third party. Self-monitoring is a type of engagement in which users can track their health behavior(s), such as step count. A review of health-related apps in the Apple Store found that self-monitoring was by far the most common form of engagement (299 out of 400 apps; 74.8%; Sama et al., 2014).

Previous research has suggested that social features complement the apps by providing an engaging platform [38]. Social media can also provide social support for quitting [39], which was found as a positive development of smoking cessation mobile apps (e.g., [27,40]. While the effectiveness of incorporating social media has not been thoroughly tested for smoking cessation, there is evidence of using social media for other health issues, such as physical activity [41], mental health (e.g., [42]), and sexual health (e.g., [43]). In Choi et al.’s [17] content analysis, researchers identified 31 smoking cessation apps that had social media capabilities but did not specify the functions or engagement strategies of the social media features, leaving the effectiveness of these capabilities unclear. Although Pechmann and colleagues showed that using Twitter in combination with a mobile device was successful in helping smokers quit, whether such a fruitful effect is generalizable across smoking cessation apps remain unknown [30].

To fill this gap in the literature, the following two research questions (RQs) were proposed.

*RQ1:* What social and non-social engagement features are present in current smoking cessation mobile apps?
*RQ2:* What social and non-social engagement features predict the engagement of smoking cessation mobile app users?

## 2. Methods

A content analysis of 149 unique smoking cessation apps was conducted in the current study. There are multiple types of content analysis. For instance, Hsieh and Shannon presented three approaches, i.e., conventional, directed, and summative, in conducting the content analysis, which primarily differ in coding schemes, origins of codes, and trustworthiness threats [44]. Given that the coding and analysis of data were guided by theories in the current study, the directed approach was followed, which is deductive and enables researchers to inform or extend existing theories [44]. To answer the research questions, descriptive statistical analyses were conducted to understand the implementation of social and non-social engagement features among the current smoking cessation apps. Furthermore, this study examined how these social and non-social features predicted the engagement of smoking cessation mobile app users using inferential statistical analyses.

### 2.1. Sampling

**Figure 1:**
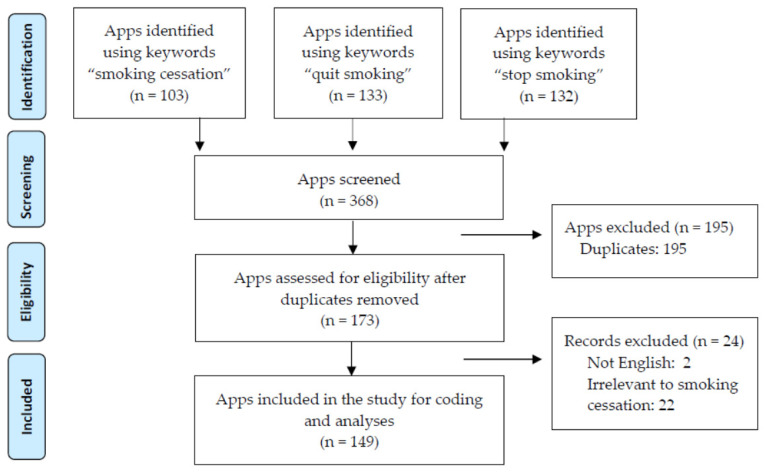
Summary of the selection process used in the study.

Smoking cessation mobile apps were collected from the Apple Store between 17th and 23rd September 2018, and were units of analysis in the current study. A portion of smoking cessation apps that are accessible from the Apple Store are also available on the Google Play Store (https://play.google.com/store/apps?hl=en (accessed on 9 July 2021)). Apps were not collected from the Google Play Store because of two reasons. First, despite using the same name in both the Apple Store and Google Play Store, the mobile apps are designed differently in iOS and Android systems, which could be a confounder for the results. Second, given that the market share of iOS (64.69%) was much larger than that of Android (35.05%) in 2018 [45], only the smoking cessation mobile apps available in Apple Store were collected. This approach is consistent with previous content analysis on smoking cessation mobile apps (e.g., [37]). Using “smoking cessation” (*n* = 103), “quit smoking” (*n* = 133), and “stop smoking” (*n* = 132) as the key terms, 368 apps were retrieved in total. To be eligible for coding, the mobile apps should be related to tobacco smoking cessation and in English. Mobile apps that were retrieved but irrelevant to smoking cessation (e.g., commercials) would introduce noise to the sample, and therefore, they were not considered. Non-English apps were excluded to ensure validity, because all coders are English-speaking. After removing the duplicated (*n =* 195), non-English (*n =* 2), and irrelevant (non-smoking cessation or pro-smoking; *n =* 22) apps, 149 unique and eligible smoking cessation apps were identified (see Figure 1 for the illustration of this process) and downloaded as the final sample of content analysis. A full list of and any specific information about the smoking cessation mobile apps collected are available upon request.

### 2.2. Coding Scheme

Besides collecting the mobile apps’ basic information, including *name*, *seller*, *price*, *version*, *age rating*, and *category*, which were provided by the Apple Store, the author and two undergraduate research assistants also coded for the (a) connection to social media (1 = *presence*, 0 = *absence*), (b) social engagement features, including *social support*, *social announcement*, and *social referencing* (1 = *presence*, 0 = *absence*) [18], (c) non-social engagement strategies, including *personal environmental changes*, *goal setting*, *progress tracking*, *reinforcement tracking*, *self-monitoring,* and *personalized recommendations* (1 = *presence*, 0 = *absence*) [18,37], and (d) user engagement. The operational definitions of each social and non-social engagement features are detailed in Table 1. Except for user engagement, all features were coded in a binary manner. 

Users’ engagement with the smoking cessation app was evaluated by the mobile app rating scale (MARS) [46], which was validated and widely applied in mHealth research to assess users’ engagement. MARS includes five questions regarding the entertainment, interest, customization, interactivity, and target group. Sample items include “Is the app fun/entertaining to use? Does it use any strategies to increase engagement through entertainment (e.g., games)?” Coders’ responses were recorded on a 5-point Likert scale ranging from 1 = *Dull, not fun or entertaining at all* to 5 = *Highly entertaining and fun, would stimulate repeat use*. The measure was reliable with the current sample (Cronbach’ *α* = 0.87).

### 2.3. Coding Procedure

To ensure that the mobile apps are coded reliably, two undergraduate research assistants, who were blind to the research objectives and questions, first independently coded ten smoking cessation mobile apps (6% of the sample) for training purposes. The two naïve coders and the researcher met to compare their coding of each mobile app. After conducting a thorough discussion to reconcile the discrepancies, the two coders independently coded another randomly selected ten apps and met with the researcher to discuss their work until reaching acceptable inter-coder reliability coefficients were calculated using Krippendorff’s *α* [47] across all key variables. A codebook, including a detailed coding instruction, was used to guide the coding process and is available upon request. The two undergraduate research assistants established satisfactory inter-coder reliability of all the coded variables: type of app (*α* = 0.76), social media connection (*α* = 1.00), social engagement features (*α* = 0.81), non-social engagement features (*α* = 0.74), and MARS (*α* = 0.80). After the inter-coder reliability was established for all variables, each research assistant independently coded half of the remaining apps. To ensure reliable coding of mobile apps, a multistage reliability check approach was applied. Once the research assistants completed coding half of the mobile apps, the researcher retrained the coders, by independently coding another ten new apps and recalculating the inter-coder reliability between the researcher and the coders. When the naïve coders had ten apps left to be coded, the procedure was repeated. By achieving satisfactory inter-coder reliability at both the mid-point and the end of the entire coding process, we were confident that the smoking cessation mobile apps in the current study had been coded reliably.

### 2.4. Data Analysis

The researcher calculated descriptive statistics for focal variables, including frequency for dichotomously coded social and non-social engagement features, and means and standard deviations for users’ engagement as an interval variable. Hierarchical multiple regressions were implemented to predict MARS with each social or non-social engagement features, adjusting for the price of mobile apps, which was found correlated with MARS score [48]. The social and non-social engagement features were analyzed individually in the regression models as the independent variable, with the MARS engagement score as the dependent variable. A *t*-test was performed to examine differences in MARS engagement scores between apps with and without connection to social media.

## 3. Results

### 3.1. Descriptive Statistics

The coded apps were predominantly commercial (96.1%), with only a few produced by the governmental (1.3%) and non-governmental organizations (0.7%) and universities (2.2%). Most apps were categorized as health and fitness (61.5%), followed by lifestyle (17.6%), medical (4.7%), and education (2.0%). Of the current smoking cessation apps, 74 (49.7%) offered tracking function, 58 (38.9%) incorporated at least a calculator (e.g., the amount of money saved by quitting smoking), 43 (28.9%) included the hypnosis, 30 (20.1%) used a calendar, and 18 (12.1%) involved an interactive game.

Regarding the social engagement features, 28.2% (*n* = 42) of the smoking cessation apps enable social announcement and 8.1% (*n* = 12) offered the social support feature. However, only two apps provided a social referencing feature (1.3%). No app included reinforcement tracking, with the percentage of other non-social engagement features ranging from 9.4% to 49.0% (see Table 1 for details).

The smoking cessation apps demonstrated a medium-level engagement score on average, with a mean score of 2.67 (*SD* = 0.72, *Min* = 1.2, *Max* = 4.8). Approximately half of the apps (48.3%) were connected to at least one social networking site (SNS), such as Facebook, Twitter, or Instagram. RQ1 was answered.

### 3.2. Inferential Statistics 

The normality, linearity, and homoscedasticity assumptions were checked prior to the implementation of regression analyses. Hierarchical regression models indicated that all three social engagement features examined in the current study—social support (*β* = 0.30, *p* < 0.001), social announcement (*β* = 0.21, *p* < 0.05), and social referencing (*β* = 0.18, *p* < 0.05)—were significant predictors of user engagement. Among the non-social engagement features, personal environment changes (*β* = 0.38, *p* < 0.001), progress tracking (*β* = 0.18, *p* < 0.05), and personalized recommendations (*β* = 0.37, *p* < 0.001) significantly predicted user engagement, while the features of goal setting (*β* = 0.14, *p* = 0.09) and self-monitoring (*β* = −0.01, *p* = 0.88) were not significant. Reinforcement tracking, absent in all the coded apps, was not analyzed. Smoking cessation apps connected to at least one SNS were more engaging compared to those not connected to any SNS (*t* (153) = 4.35, *p* < 0.001). RQ2 was addressed (see Table 1 for the summary of descriptive and inferential statistical results).

## 4. Discussion

The increasing popularity of mobile devices among the U.S. adults provided an unprecedented opportunity to promote smoking cessation via mobile apps, which offer a variety of interactive and customizable tools conducive to quitting smoking [4]. However, the low engagement has always been the primary concern for the effectiveness of mobile phone-based smoking cessation interventions [30]. It was found in the current study that the mean engagement score (*M* = 2.67) of mobile apps in the U.S. was lower than the mean score documented in the sample of French apps (*M* = 3.5), which was based on 14 apps though [48]. By conducting a comprehensive content analysis of free and paid smoking cessation apps, this study answered two primary research questions: (a) what social (and non-social) engagement features are present in smoking cessation apps and (b) how effective they are in potentially engaging users. The findings not only applied and extended the mobile health app engagement typology [18], but provided practical guidance to smoking cessation apps design.

### 4.1. Social Engagement Features

The current analysis showed consistency with previous studies [17,28] that the smoking cessation apps primarily applied basic features, such as calculator and calendar, but have not fully explored the social features, given their low frequencies, to stimulate autonomous motivation. Specifically, among the 149 coded mobile apps, only 2 (1.3%) included the social referencing feature. Although a friendly competitive environment was suggested as helpful in increasing engagement by scholars [49,50], which found empirical support in previous mHealth literature [51] and the current study, the result could be by chance given the small sample size. Even so, the finding still indicated the potential of engaging smokers in the quitting process by creating comfortable competition among them in mobile apps.

The effectiveness of a supportive feature in engaging smokers identified in the current study echoes to existing mobile app-based health behavior change literature (e.g., [52]). The three major types of social support—emotional, informational, and instrumental—could all be provided either generally or “abstinence specific” [40] (p. 698). For instance, the app *Craving to Quit!* built a community for users, where the smokers are able to vent their frustration and encourage each other to overcome the difficulty in the process of abstaining, which are considered as abstinence-specific emotional support, as well as provide information about quit line or nicotine replacement therapy to another smoker as abstinence-specific information support [40]. Online social support groups, which were documented to have a significantly positive effect on health outcomes [53], could be particularly promising for smoking cessation via mobile apps. Given the addictive nature of smoking behavior, the cessation is particularly challenging and usually a long-term process, which explains the low abstinence rate among self-quitters [54]. However, the stress-buffering model [32] rationalizes the process in which social support attenuates the intensity of smokers’ stress, and therefore, users are more likely to be engaged with the smoking cessation apps.

Despite the mixed empirical evidence of social announcement or presentation in improving health outcomes [51,55], it was found positively associated with perceived engagement. Users generally enjoy the process of announcing their achievements to their social networks, which enhances their competence and self-efficacy, serving as factors to induce intrinsic motivation in this effortful abstaining process. However, the current finding was at odds with the negative moderating effect of social announcement feature documented in a meta-analytic review of mobile phone-based interventions in improving health outcomes [3]. It should be noted that users’ engagement with the mobile app, albeit necessary for behavior change and improving health outcome, may not be enough by itself to trigger the process of positive change. Therefore, the strategies of bridging the engagement with the device per se and the downstream health outcome improvement is an equally important link in this mobile app-based behavior smoking cessation chain.

That the smoking cessation apps connected to at least one SNS were more engaging than those not connected to any SNS could be attributed to the overlaps between SNS connection and the incorporation of social engagement features. The majority of smoking cessation apps that included a social support (10/12), social announcement (39/42), and social referencing features (1/2) were connected to SNSs, the built-in social networking functions of which were taken advantage of by the mHealth app designers. Thus, future mHealth apps are recommended to integrate links of external SNSs into the health-related functions in apps, to facilitate social supportive and announcing interactions between users and their social ties.

### 4.2. Non-Social Engagement Features 

That *personal environment changes* and *personalized recommendations* features were positively associated with the engagement score is in line with Abroms and colleagues’ [23] findings from *Text2Quit*, a personalized and interactive mobile health program, and the qualitative evidence gleaned by Perski et al. [56] using semi-structured interviews. Receiving text messages tailored around each participant’s demographic (i.e., name and gender) and quitting-related information, including chosen quit date, reasons for quitting, estimated money saved, and person selected for social support, the participants reacted positively towards this program [23]. As one of the additional advantages of mobile apps compared to the text messaging, customizable features were also found effective in helping smokers in the quitting process [7] and superior than generic messages in changing health behaviors [57]. The theoretical explanation for the positive effects of personalized environment changes and recommendations in engaging smoking cessation mobile app users was provided by the elaboration likelihood model (ELM) [58], which suggested that when processing a persuasive message, individuals may go through a *central route*, where they carefully examine the arguments, or a *peripheral route*, where a mental short cut or heuristic cues are applied. According to ELM, tailored feedback and interface, perceived as personally relevant, enhance individuals’ motivation to elaborate the messages, and therefore, trigger the central route processing, which is more likely to engage the mobile app users.

Although setting a quitting goal by itself was not a significant predictor, enabling users to track their progress towards their goals turned out to be a significantly predictive factor of engagement. Tracking progress was also found to be one of the most accepted and utilized features in a systematic review of smoking cessation mobile apps [59]. According to the social cognitive theory [34,35], assessing one’s behavior against the goal serves as an important process of self-regulation. By tracking and comparing progress towards identified smoking cessation goals, the app users could adjust their behaviors to reach the goals, which leads to increased self-efficacy and engagement in this quitting process. In the same vein, the social determination theory explains that functions such as recording and tracking one’s progress enhance smokers’ sense of competence, which is necessary for their intrinsic motivation [36].

### 4.3. Theoretical and Practical Implications 

Previous research has suggested that social features complement the apps by providing an engaging platform [38], and provide crucial components (e.g., social support) of successful smoking cessation interventions [40]. Despite the evidence of using social features for physical activity [41], mental health [42], and sexual health [43], the effectiveness of social features has not been thoroughly tested for smoking cessation, which leaves unclear which specific forms of social engagement features (i.e., social support, social announcement, and social referencing) lead to higher engagement and effectiveness. Above and beyond depicting the landscape of engagement features applied by existing smoking cessation mobile apps, the current study identified the effectiveness of engaging smokers by theory-based features, including social support (e.g., encouraging, congratulating, and comforting), friendly competition (e.g., listing the top users based on their cessation days), progress tracking, and personalization, showing consistency with previous studies [13,60]. The identification of effective engagement features not only lend support to the applicability of buffering hypothesis [32], downward social comparison [33], social cognitive theory [34,35], self-determination theory [36], and ELM [58] in smoking cessation, but also extend these theoretical frameworks to the mobile context.

The findings also contributed to the mHealth scholarship by extending Sama et al.’s typology [18] to the smoking cessation context. Among the first studies that closely investigated the characteristics of the health care apps available in the Apple Store, the nine engagement categories—(1) changing personal environment, (2) facilitating social support, (3) goal setting, (4) progress tracking, (5) reinforcement tracking, (6) self-monitoring, (7) social presentation or achievement, (8) social referencing, and (9) other—have been widely applied in a variety of health topics. However, it is surprising that even though tobacco smoking remains the leading cause of preventable death in the U.S and claims over 480,000 lives every year [5], no study has yet examined the engagement features in smoking-related apps. Smoking cessation mobile apps have been overlooked not only by scholars, but also designers, as the most popular mHealth apps have focused on fitness and self-management [18]. Therefore, by innovatively analyzing smoking-related mobile apps and identifying the effective engagement features under Sama and colleagues’ framework [18], this study filled the gap of our knowledge about how to engage smokers using mobile devices and draw scholars’ attention to leveraging mobile persuasive technology to discourage the use of tobacco products and other substances.

The study also provides practical guidance to the design of future smoking cessation mobile apps. First, compared to the basic features, such as information, calculator, calendar, and sophisticated social engagement features, despite their promise in engaging users, are still underused, especially the social supportive and referencing features. Despite the caveat of not overcomplicating the mobile apps to prevent overwhelming or confusing users, as complex applications are unnecessary for motivating real behavior change [49], designers could consider incorporating one or two social engagement features to enable smokers to go through this challenging quitting process with peers sharing similar experience and characteristics. Second, basically setting a quitting goal or monitoring tobacco use turned out not to be highly engaging to app users, whereas enabling users to track progress against their goal increases their sense of autonomy and competence, which further boost their intrinsic motivation [36]. Therefore, the mobile app designers, after collecting information about users’ smoking behavior and quitting goals, should make sure to enhance users’ self-efficacy and engagement, by providing tailored feedback based on users’ progress and making them trackable on the apps. Finally, considering that people generally favor positive reinforcement, smoking cessation apps are suggested to include rewards for periodical achievements for users, especially because quitting is a long-term and arduous process, in the middle of which a large number of former smokers’ relapse. Thus, how to reinforce smokers progress, even if it is trivial, and fuel them to move to the next quitting stage through the affordances available on interface is crucial for mobile app design.

### 4.4. Limitation and Future Research

There are several limitations of the study that should be noted. First, for the comparability of smoking cessation apps, the current study only examined apps in the Apple Store, but not in the Google Play store. Although a large number of apps have both versions, there may exist some apps that were only developed on the Android system. Second, although this project started from a comprehensive search, certain features were sparsely present in the current sample (e.g., social referencing and reinforcement tracking), which limits the interpretability of the inferential statistics. Given the theoretical foundations, future mobile apps could incorporate features that enable users to compare their progress with other users in a friendly competitive environment and involve a third party to assign reinforcements based on users’ health behavior and outcome information. Third, since self-reported measures could be biased [61], the MARS [46] was applied to evaluate the engagement score of smoking cessation apps generated by coders. Although the MARS was a validated scale to evaluate mobile apps and the inter-coder reliability was established, it might still be subjective and different from the engagement reported by actual users. Future research is suggested to examine user engagement using multiple approaches, such as recruiting and training a large number of mobile app users to assess the apps. Finally, despite the identification of social and non-social features that potentially contribute to user engagement, the cognitive and psychological mechanism of such effects remain unclear using the content analytic approach. Future research is encouraged to assess potential mediators and moderators of relationships between the specified features and engagement, which could provide an even more informative account of why a particular function or dimension of social features is effective and for whom it is effective [40].

## 5. Conclusions

Although engagement and user-friendliness of an app do not necessarily ensure its effectiveness for smoking cessation [37], engagement has been identified as a key factor in predicting the effectiveness of mobile apps for health interventions. Electronic technologies, which include interactive components, such as social media, promote engagement through social connections [62]. To the best of our knowledge, this is among the first studies that systematically examines social and non-social engagement features in smoking cessation apps using content analytic approach, and extended previous content analyses (e.g., [17,37]) by investigating which social and non-social features are more likely to engage users. The findings of the engagement features’ prevalence and effectiveness in engaging smokers lend support to the theoretical frameworks underlying the engagement features and shed light on the mobile health apps engagement typology. Furthermore, valuable guidance was provided for future architecture design of persuasive technology using mobile devices, such as emphasizing the customized interface and recommendations and facilitating supportive and comfortably competitive environments among users.

## Figures and Tables

**Table 1:** Definition, frequency, and inferential statistics of engagement features.

Engagement Features		Operational Definition	*n* (%)	DV: Engagement
**Social**	1. Social support	The app has or creates groups for the users to interact with online or in person for discussing stress, problem solving, encouraging, etc.	12 (8.1)	*β* = 0.30 ***
	2. Social announcement	The app allows the user to implicit social reinforcement through announcing an achievement, process, or action through an app tool or social media.	42 (28.2)	*β* = 0.21 *
	3. Social referencing	The app allows the user to compare their behavior (must be quantifiable such as user score) to other app users, such as through an online or social group that uses the same feature.	2 (1.3)	*β* = 0.18 *
**Non-social**	1. Personal environmental changes	The environment in the app changes in response to user’s action to promote smoking cessation through the use of soothing sounds, images for meditations, white noise, or other changes.	59 (39.6)	*β* = 0.38 ***
	2. Goal setting	The app promotes goal setting, such as setting the number of cigarettes that will be decreased day.	34 (22.8)	*β* = 0.14
	3. Progress tracking	The app allows users to identify a goal and then creates tasks based on these goals and tracks the user’s progress.	31 (20.8)	*β* = 0.18 *
	4. Reinforcement tracking	A third party assigns reinforcements based on information collected about the user’s health or behaviors.	0 (0.0)	-
	5. Self-monitoring	The app allows the user to track their behavior (e.g., number of cigarettes smoked per day) with no reference to a specific goal.	73 (49.0)	*β* = −0.01
	6. Personalized recommendations	The app provides tailored verbal/textual detailed feedback and responses based on relevant questions. Examples include tips and messages based on the user’s needs. Does not include tracking.	14 (9.4)	*β* = 0.37 ***

*Note.* Definitions were adapted from Sama et al. [18] and Ubhi et al. [37]. * p < 0.05. ** p < 0.01. *** p < 0.001.

## Data Availability

The data presented in this study are available on request from the corresponding author.
